# Fano resonance Rabi splitting of surface plasmons

**DOI:** 10.1038/s41598-017-08221-5

**Published:** 2017-08-14

**Authors:** Zhiguang Liu, Jiafang Li, Zhe Liu, Wuxia Li, Junjie Li, Changzhi Gu, Zhi-Yuan Li

**Affiliations:** 1 Institute of Physics, Chinese Academy of Sciences, Beijing, 100190 China; 2 Collaborative Innovation Center of Quantum Matter, Beijing, 200092 China; 3 College of Physics and Optoelectronics, South China University of Technology, Guangzhou, 510640 China; 4 University of Chinese Academy of Sciences, Beijing, 100049 China

## Abstract

Rabi splitting and Fano resonance are well-known physical phenomena in conventional quantum systems as atoms and quantum dots, arising from strong interaction between two quantum states. In recent years similar features have been observed in various nanophotonic and nanoplasmonic systems. Yet, realization of strong interaction between two or more Fano resonance states has not been accomplished either in quantum or in optical systems. Here we report the observation of Rabi splitting of two strongly coupled surface plasmon Fano resonance states in a three-dimensional plasmonic nanostructure consisting of vertical asymmetric split-ring resonators. The plasmonic system stably supports triple Fano resonance states and double Rabi splittings can occur between lower and upper pairs of the Fano resonance states. The experimental discovery agrees excellently with rigorous numerical simulations, and is well explained by an analytical three-oscillator model. The discovery of Fano resonance Rabi splitting could provide a stimulating insight to explore new fundamental physics in analogous atomic systems and could be used to significantly enhance light-matter interaction for optical sensing and detecting applications.

## Introduction

**Figure 1:**
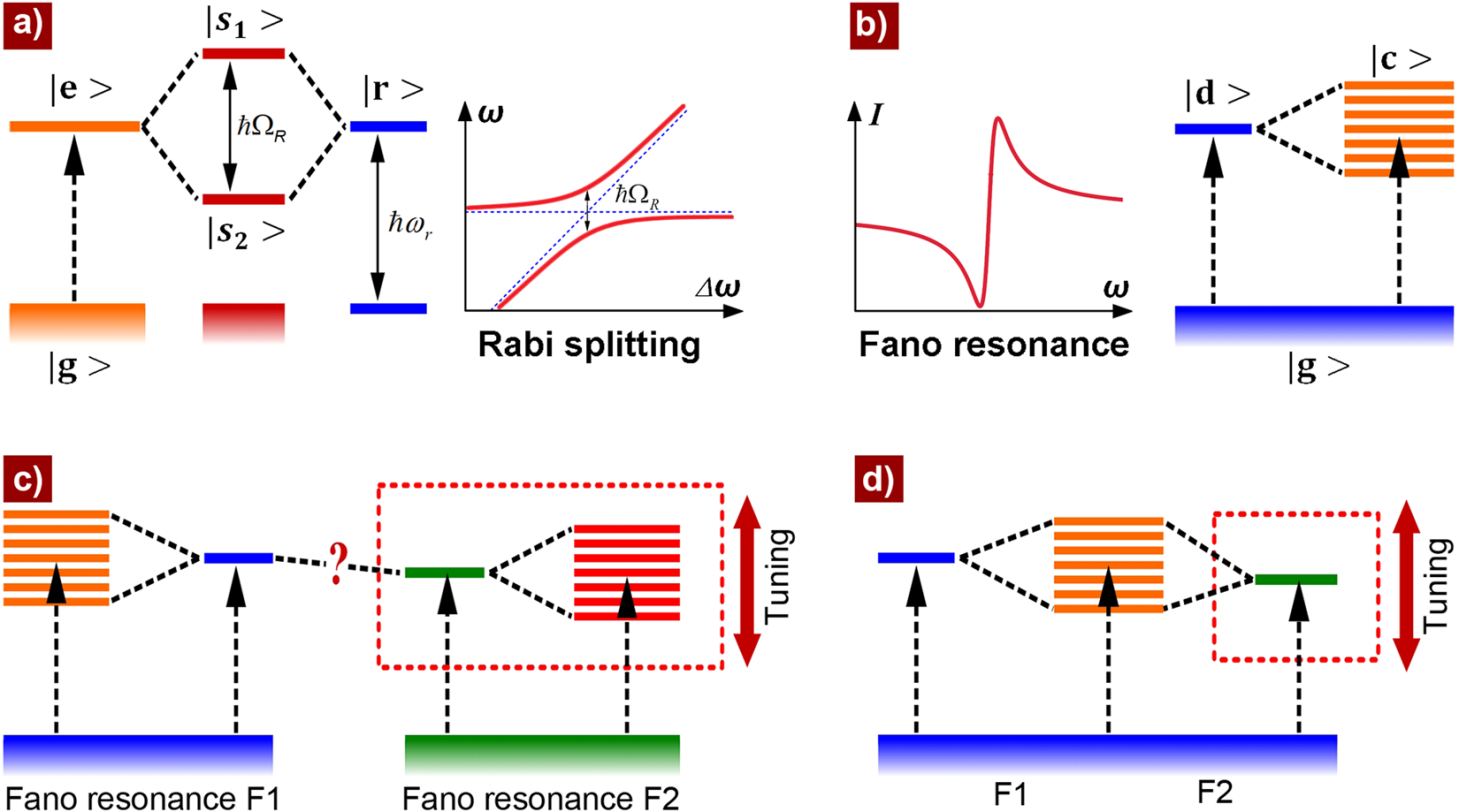
Rabi splitting and Fano resonance. (**a**) Schematic illustration of the Rabi splitting. State $|{\rm{e}} >$ (excitation states of atoms, molecules, quantum dots, plamons, etc.) strongly couples with resonance state $|{\rm{r}} >$ (i.e., cavity resonance, plasmonic resonance, etc.) of frequency ${\omega }_{r}$, which causes the levels repelled by a Rabi splitting of $\hslash {{\rm{\Omega }}}_{R}$. (**b**) Schematic illustration of the Fano resonance. The interference between a discrete state $|{\rm{d}} >$ and a continuum $|{\rm{c}} >$ results in the Fano resonance with asymmetric line shape. Both the discrete states and the continuum could be multiple according to Fano’s initial framework^20^. (**c**) Schematic illustration of the possibly existing strong coupling between two Fano resonances if both the discrete state and the continuum of one Fano resonance (for example, F2 as enclosed by the dashed rectangle) could be tuned simultaneously across the other. (**d**) Schematic illustration of the simplified scheme for possible strong coupling between two Fano resonances that share the same continuum state. In such a case, strong interaction between the two Fano resonances may occur by simply varying the discrete state of one Fano resonance (as enclosed by the dashed rectangle). The possibility and results of the strong interaction occurring in Fig. 1c,d have yet been explored.

Vacuum Rabi splitting, originally describing the large anticrossing between the atom-like emitter and cavity-mode dispersion relations (Fig. 1a), is an important phenomenon that has been of great interest in atomic physics since 1980s^1–4^. Compared with normal mode couplings, Rabi splitting refers in particular to strong coupling in which the coupling strength exceeds the dissipation rates of the system and the energy is therefore coherently exchanged between atom and cavity photon. This enables quantum coherent oscillations between the coupled systems and the quantum superpositions between different quantum states, which are essentially important for quantum information processing^2, 3, 5, 6^. Therefore, Rabi splitting has been extensively investigated in various quantum and semi-classical systems, such as quantum-dot-microcavity^2, 3, 5, 6^ and emitter-plasmon systems^4, 7–12^, which was studied not only in emission but also in scattering, absorption, transmission or reflection properties. More recently, the quantization of plasmons^13–15^ extends the concept of Rabi splitting more generally into the emerging plasmon-cavity and plasmon-plasmon coupling systems beyond the traditional emitter-field context^4, 16–19^. As another important phenomenon in atomic physics, Fano resonance was recognized even earlier in 1961 by Ugo Fano to describe the quantum interference between a discrete excited autoionized state and a continuum (Fig. 1b)^20^. Featured by its asymmetric line-shape and steep profile, Fano resonance has also been widely extended from atomic system to photonic crystals, metamaterials, and other plasmonic systems^21–23^, with potential applications in ultrasensitive biosensing, high-contrast optical imaging, high-quality optical waveguides, etc^21, 24^. Yet, the realization of strong interaction between two or more Fano resonance states (Fig. 1c), allowing for coherent manipulation of multiple complex or remote quantum states that could be used in principle as simulators for quantum many-body and quantum field theory systems^4, 25^, has not been accomplished either in atomic or in optical systems.

**Figure 2:**
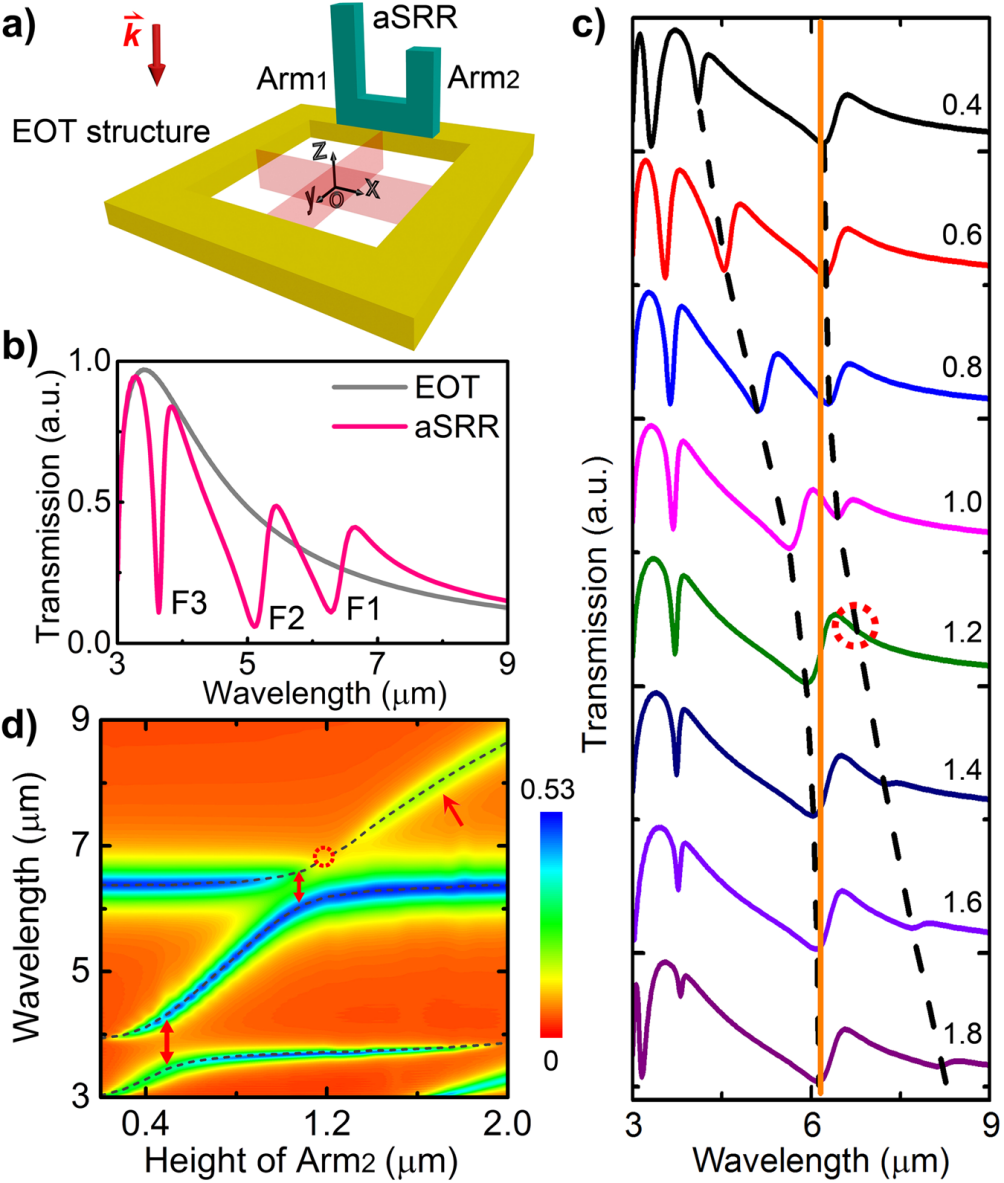
Triple Fano resonances and double Rabi splittings. (**a**) Schematic of the proposed substrate-free 3D structure made of gold, which consists of vertical asymmetric SRRs (with arms of Arm_1_ and Arm_2_) standing along one edge of the metallic hole array with EOT properties. (**b**) Simulated transmission spectra of an EOT structure and the proposed 3D structure with arm height of H_Arm1_ = 1.2 µm and H_Arm2_ = 0.8 µm. Y-polarized light excitation is used throughout this work. Triple Fano resonances noted by F1, F2, and F3 are clearly observed. (**c**) Simulated transmission spectra of the 3D structure with H_Arm1_ = 1.2 µm and different H_Arm2_ as noted. The dashed curves and vertical straight line (guides to the eye) clearly reflect the anticrossing behavior between Fano resonance F1 and F2 (the anticrossing between Fano resonance F2 and F3 is manifested in Supplementary Fig. S3). This anticrossing in multiple Fano resonances has not been seen in any uncoupled Fano resonant systems^26, 30^. (**d**) Colormap of the simulated absorption spectra of the proposed structure versus the height of Arm_2_. The Fano resonance wavelengths as plotted by the dashed curves are identified more clearly in absorption spectra (Supplementary Fig. S1). The red double-head arrows indicate the position of F1-F2 (above) and F2-F3 (below) anticrossings, of which the Rabi splitting energy is ~17 and ~76 meV, respectively, in contrast with the resonance energy of ~190 and ~320 meV. The red single-head arrow points out the recovery of the diminished branch of the upper band during F1-F2 anticrossing. The dashed circles in Fig. 2c,d indicate the singularity in F1-F2 anticrossing when H_Arm1_ = H_Arm2_ = 1.2 μm. Structural parameters (defined in Supplementary Fig. S1): lattice periodicity *p* = 3 µm, square hole width *a* = 2 µm, Au film thickness *d* = 80 nm, width of SRR *l* = 1 µm, and SRR arm width *w* = 0.25 µm.

The challenge of achieving strong coupling between two Fano resonance states in a single system lies in the difficulty of tuning both the discrete state and the continuum of one Fano resonance simultaneously without the influence of the other (Fig. 1c). However, considering the unique feature of Fano resonances, the complicated four-body state interaction between two Fano resonance states could be simplified into a three-body issue if the two Fano resonance states share the same continuum, as shown in Fig. 1d. In such a case, to achieve the strong interaction between two Fano resonances, one needs to tune only one of the discrete states (Fig. 1d). Based on this scheme and inspired by our previous work with double Fano resonances in a symmetric structure^26^, here we propose a symmetry-broken three-dimensional (3D) plasmonic structure with triple Fano resonances that share the same “continuum” (Fig. 2a,b), i. e., the proposed plasmonic system possesses three fundamental “discrete” states but one “continuum” state, of which the interferences result in significant triple Fano resonances. Through tuning one of the Fano resonances across the other two by varying one of three discrete states, double Rabi splittings in Fano resonances are observed. Actually, such an important system related with multiple discrete states and one continuum has been theoretically mentioned by U. Fano in 1961^20^, which, however, has not been realized in atomic or optical systems due to its complexity and difficulty, not to mention the inter-coupling among the multiple Fano resonance states. We show here that the introduction of asymmetry, with which the structural modification looks somehow “simple” at the first sight, could provide a novel and effective approach for the engineering of interaction among multiple “discrete” and “continuum” states, implementing the one of earliest proposals from U. Fano^20^.

## Results

### Double anticrossings in triple Fano resonances

As schematically illustrated in Fig. 2a, the proposed 3D structure is formed by integrating vertical asymmetric SRRs (aSRRs) along a planar air hole array with extraordinary optical transmission (EOT)^27^. It is well-known that in SRRs the electric resonances could be excited by E-field parallel to the two arms (i.e. z-polarized light in Fig. 2a) and the magnetic resonances can be excited by E-field perpendicular to the two arms while parallel to the SRR-plane (i.e. x-polarized light in Fig. 2a)^28, 29^. However, due to a 3D conductive coupling mechanism^30^, it is only under y-polarized light excitation that significant Fano resonances could be driven by z-component of the plasmonic field along the planar hole arrays. Therefore, y-polarized light excitation is mainly used in this paper, with respect of which the symmetry of the structure is broken when the two arms of the aSRR structure have different height. As shown in Fig. 2b, by setting the height of aSRR arms with H_Arm1_ = 1.2 μm and H_Arm2_ = 0.8 μm, prominent triple Fano resonances (named as F1, F2 and F3) can be generated in the plasmonic system (Supplementary Fig. S1). The triple Fano resonances are associated with different current oscillations via 3D conductive coupling, which will be discussed in the following. It should be emphasized that compared with the independent double Fano resonances in a symmetric structure, the broken symmetry induced Fano resonances in this 3D plasmonic configuration offer unique advantages in engineering the interaction among multiple Fano resonances. For instance, Fano resonance F2 can be readily tuned (Supplementary Fig. S2) across Fano resonances F3 and F1 by simply varying the height of one arm of the aSRRs (H_Arm2_ from 0.4 to 1.8 µm), as shown in Fig. 2c. Importantly, double anticrossings (F1-F2 and F2-F3 anticrossings) are clearly observed during this tuning process, as illustrated in Fig. 2c (also in Supplementary Fig. S3) and Fig. 2d. These double anticrossing behaviors are very different from the normal mode crossing, which does not involve coupling process although asymmetry could induce splitting and shift in spectra^31^. The ratios of the splitting and the linewidth for F2-F3 and F1-F2 anticrossings are 3.30 and 1.03, respectively, both of which meet the criterion of strong couplings^16^ and therefore validate the double Rabi splittings in Fano resonances. It should be mentioned that according to Fig. 1d, the position of the fixed Fano resonance (F1, corresponding to H_Arm1_) determines the Rabi splitting region. This characteristic is also proved in our plasmonic system by that the F1-F2 anticrossing region can be tuned by varying the Fano resonance F1 (i.e., by simply changing H_Arm1_ as illustrated in Supplementary Fig. S4). Particularly when H_Arm1_ = 0, the F1-F2 anticrossing disappears and the double anticrossings degrade into a single anticrossing (F2-F3 anticrossing in Supplementary Fig. S4).

It is worthwhile to point out two features in Fig. 2d. Firstly, there is a singularity right in the middle of the upper band of the F1-F2 anticrossing, as indicated by the dashed circle. This feature is also reflected in Fig. 2c where the triple Fano resonances degenerate into double Fano resonances when both arm heights of the aSRRs are 1.2 µm (H_Arm1_ = H_Arm2_ = 1.2 µm, as noted by the dashed circle). This singularity, together with the diminishment of half of the upper or/and lower anticrossing band, did actually exist in many reported plasmon-plasmon strong coupling schemes^4, 16, 18, 32, 33^ and other mesoscopic systems^25^, but their physical mechanism has not been clarified. Here we attribute this singularity to the degeneracy of the modal asymmetry, which is required by the classical anticrossings (see refs 34, 35 and following discussions). In comparison, there is no singularity in the F2-F3 anticrossing since the structural asymmetry is preserved during the tuning process. The second feature of the F1-F2 anticrossing is the recovery of the diminished branch of the upper band, as noted by the red arrow in Fig. 2d, which is very different from conventional plasmon-plasmon strong coupling where no recovery was observed. This feature is benefited from the unique tunability of the proposed 3D structures in that when the arm height (H_Arm2_) is changed to larger values, the asymmetry of the mode coupling is recovered and so is the anticrossing process.

### Harmonic oscillator modeling

**Figure 3:**
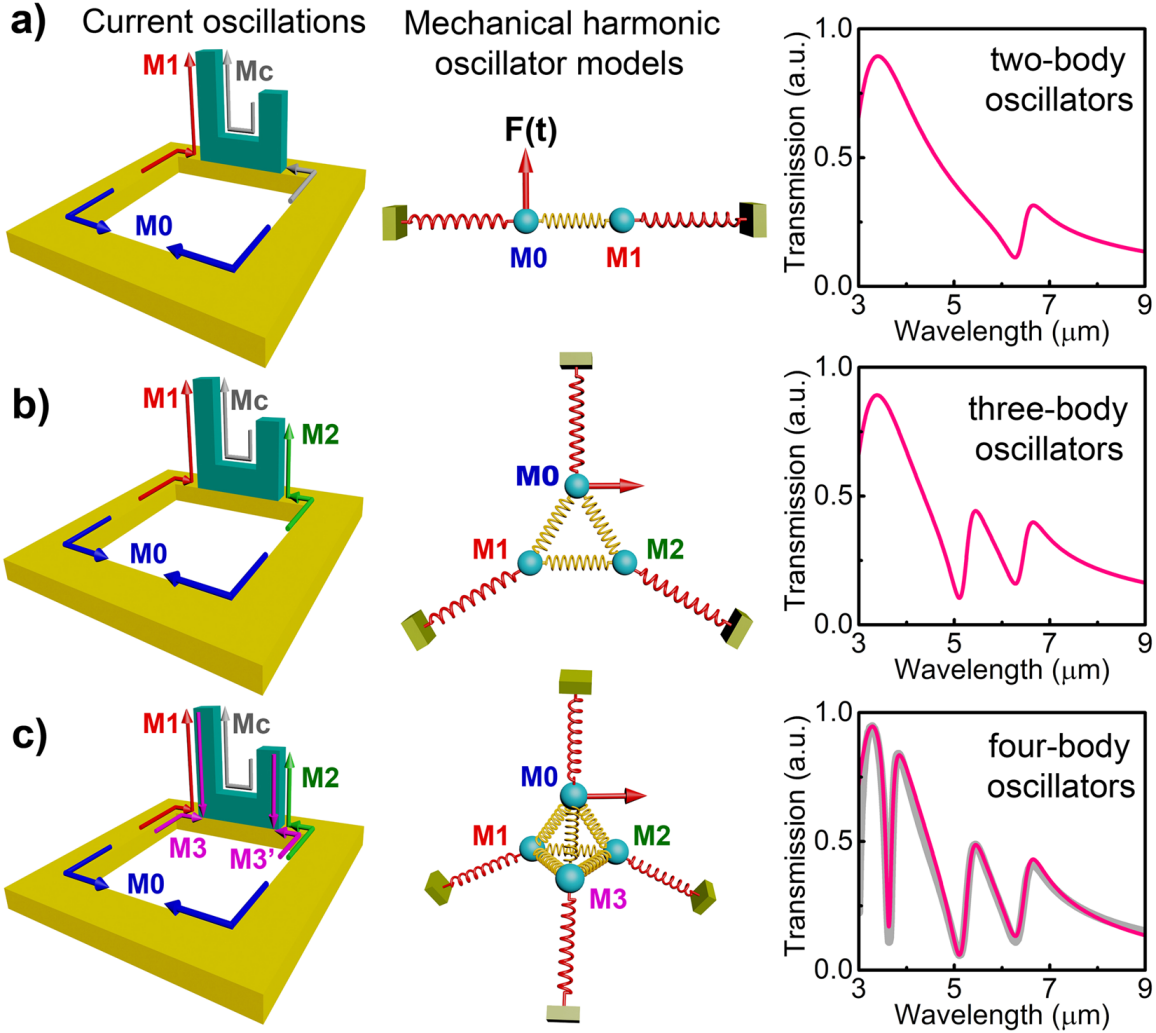
Oscillator modeling. (left) Schematic plots of the oscillating currents under different resonant mode couplings, (center) the mechanical harmonic oscillator models, and (right) the analytically calculated transmission of the 3D structure with arm height of H_Arm1_ = 1.2 µm and H_Arm2_ = 0.8 µm, in the models of (**a**) two-body oscillators, (**b**) three-body oscillators, and (**c**) four-body oscillators, respectively. The circular current flows (Mc) in the left column provide an interaction channel to mediate the charge density along the two arms. The grey spectra in Fig. 3c represents the plot of the numerical result in Fig. 2b, which is well consistent with the analytical calculation. In all three models, k_12_ = k_13_ = k_23_ = 0 is used since in this structure, the Fano resonances are far away from the anticrossing regions and therefore not coupled. Other coupling coefficients are employed as in Supplementary Section II. Structural parameters are the same as in Fig. 2. From these models, it can be seen that Fano resonances F1, F2 and F3 result from the M0-M1 (interference between modes M0 and M1), M0-M2, and M0-M3 interferences, respectively (Supplementary Fig. S6).

One characteristics of the proposed 3D plasmonic system is that the origins of all the triple Fano resonances are involved with the same continuum, i.e. the triple Fano resonances result from the interference between one broad resonance from EOT structure and three discrete resonances from conductive coupled plasmonic modes, respectively. It should be noted that multiple Fano resonances, resulted from the coherent coupling between superradiant and subradiant plasmon modes, have been observed in the dolmen-style slabs and ring/disk dimers without the study of the coupling among subradiant modes^36^. In our case, the plasmonic system could simplify the interaction among three Fano resonances from a complicated six-body system into a four-body case, which could be analyzed by using a mechanical model consisting of four harmonic oscillators (Supplementary Section II)^37, 38^, as illustrated in Fig. 3. In this model, the four oscillators influence each other through six springs with coupling coefficients of *k*_01_, *k*_02_, *k*_03_, *k*_12_, *k*_13_ and *k*_23_, respectively, in which *m*_1_ = *m*_2_ = *m*_3_ = *m*_4_ = 1 is set for simplicity. In our case, an input harmonic force F(t) = Ae^−iωt^ is employed to drive the oscillator $|{\rm{M}}0\rangle$, which is analogous to the y-polarized light excitation of the EOT resonance. Oscillators $|{\rm{M}}1\rangle$,$|{\rm{M}}2\rangle$, and $|{\rm{M}}3\rangle$ are treated as subradiant modes (Supplementary Fig. S6) that cannot be directly excited by incident light polarized along x-/y-direction. However, they can be induced by the surface plasmons with electric field along z-direction excited at the metal-dielectric interfaces.

To identify the origins of the initial triple Fano resonances without interactions, we firstly analyze a structure with H_Arm1_ = 1.2 µm and H_Arm2_ = 0.8 µm that supports triple Fano resonances far away from the anticrossing regions, i.e. the triple Fano resonances are relatively independent. In such a specific structure, one obtains k_12_ = k_13_ = k_23_ = 0. If one sets k_02_ = k_03_ = 0 and k_01_ ≠ 0, the system is then simplified into a two-oscillator model in the middle of Fig. 3a, with which the Fano resonance F1 could be represented by a two-state coupling between the broad EOT resonance M0 and a narrow resonance M1 (left of Fig. 3a), as the calculated spectrum shown in the right of Fig. 3a. Similarly, in the case of k_03_ = 0, k_01_ ≠ 0, and k_02_ ≠ 0, a three-oscillator system is constructed, which generates double Fano resonances (F1 and F2) due to the introduction of the mode M2 associated with the short arm of the aSRRs (Arm_2_), as shown in Fig. 3b. In the realistic case, k_01_, k_02_, and k_03_ are all non-zero and the plasmonic system should be described by a four-oscillator system that induces triple Fano resonances F1, F2 and F3, as manifested in Fig. 3c. It should be mentioned that the calculated spectrum with this four-oscillator model theory (right of Fig. 3c) agrees very well with the numerical calculation in Fig. 2b, indicating the excellent accuracy of our oscillator models and the factual couplings among different modes (left of Fig. 3c). The “evolution” of Fano resonances in Fig. 3a–c clearly manifests the origins of all the three Fano resonances in the aSRR-based plasmonic system (Supplementary Fig. S6): Fano resonance F1 results from the interference between M0 and M1; Fano resonance F2 is formed due to the coupling between M0 and M2; the interaction between M0 and M3 results in Fano resonance F3. In a word, for all the three Fano resonances, the broad EOT resonance mode M0 serves as a shared superradiant mode, providing energy to couple with the other three subradiant modes respectively, which is the unique feature of the 3D plasmonic system.

Now that we have identified the origin of the triple Fano resonance states, we proceed to see what interesting would happen when these initial states are brought together via mutual electromagnetic coupling due to close spatial and spectral overlapping. However, when the initial modes M1, M2 and M3 get coupled, k_12_, k_13_ or k_23_ is nonzero. Moreover, when M2 is continuously tuned by varying H_Arm2_, each value of H_Arm2_ introduces three different coupling coefficients k_02_, k_12_, and k_23_. These extra coupling factors introduces great difficulties in finding nontrivial solution and eigenvalues of the inhomogeneous linear differential equation that describes the four-oscillator system [Eq. (2) of the Supplementary Information]. Alternatively, this complicated interaction could be mathematically simplified into a secondary interaction among the initial triple Fano resonances, i.e. the interaction merely among initial Fano resonances F1, F2 and F3 (Supplementary Section III). When the triple Fano resonances are closely coupled, their interaction could be simply described by a homogeneous linear differential equation that is similar to a three-oscillator expression (Supplementary Section III)^37^, as

1 $$(\begin{array}{ccc}-\,{\omega }^{2}-i{s}_{1}\omega +{f}_{1}^{2} & -{{\rm{\Lambda }}}_{12} & -{{\rm{\Lambda }}}_{13}\\ -{{\rm{\Lambda }}}_{12} & -{\omega }^{2}-i{s}_{2}\omega +{f}_{2}^{2} & -{{\rm{\Lambda }}}_{23}\\ -{{\rm{\Lambda }}}_{13} & -{{\rm{\Lambda }}}_{23} & -{\omega }^{2}-i{s}_{3}\omega +{f}_{3}^{2}\end{array})(\begin{array}{c}{y}_{1}\\ {y}_{2}\\ {y}_{3}\end{array})={\rm{M}}\cdot (\begin{array}{c}{y}_{1}\\ {y}_{2}\\ {y}_{3}\end{array})=(\begin{array}{c}0\\ 0\\ 0\end{array})$$

where *y*_*i*_ = *Qe* ^−*iωt*^ (i = 1, 2, and 3) represents the resulted resonances, *s*_*i*_ is the friction coefficient, *f*_*i*_ is the frequency of the initial Fano resonance, Λ_*ij*_ is the coupling coefficient between Fano resonances *f*_*i*_ and *f*_*j*_, and M is the coefficient matrix of the equation.

**Figure 4:**
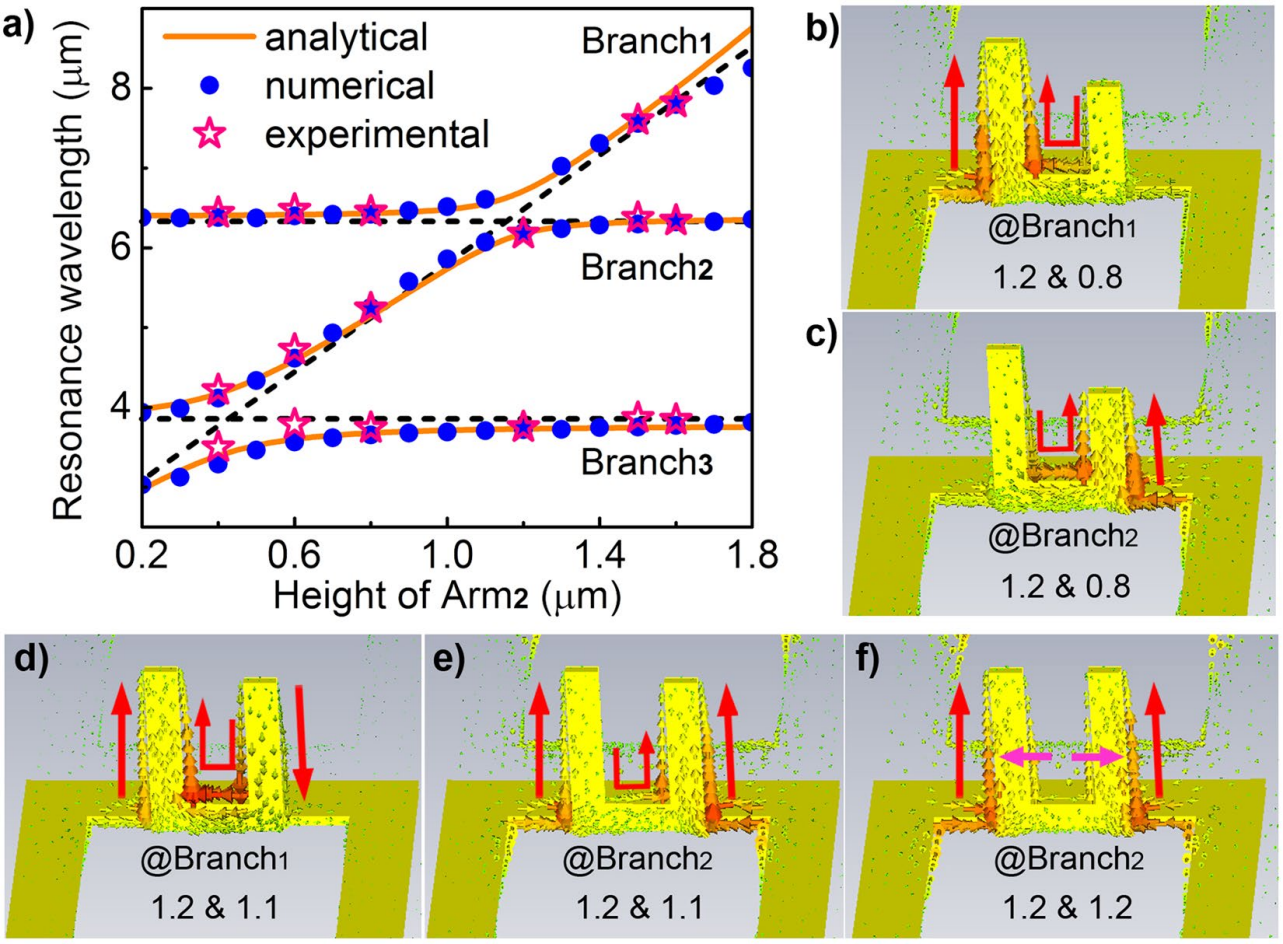
Analysis and discussions. (**a**) Relationship between the resonance wavelength and the height of Arm_2_ under numerical simulations (blue dots), analytical calculations (orange lines), and experimental measurements (red stars). H_Arm1_ = 1.2 µm. (**b**,**c**) Simulated current distributions of the aSRRs with H_Arm1_ = 1.2 µm and H_Arm2_ = 0.8 µm at corresponding resonance wavelengths in (**b**) Branch_1_ and (**c**) Branch_2_, respectively. It can be seen that in this structure Branch_1_ resonance is mainly dependent on the current oscillation along Arm_1_ (the current flow along Arm_2_ is negligible) while Branch_2_ resonance mainly relies on the current oscillation along Arm_2_ (the current flow along Arm_1_ is negligible), as schematically noted by the red arrows. (**d**,**e**) Simulated current distributions of the structure with H_Arm1_ = 1.2 µm and H_Arm2_ = 1.1 µm at corresponding resonance wavelengths in (**b**) Branch_1_ and (**c**) Branch_2_, respectively. It can be seen that in the anticrossing region both arms of the SRRs take part in electric current interaction through the circular current flows. (**f**) Simulated current distributions of the structure with H_Arm1_ = H_Arm2_ = 1.2 µm at the Branch_2_ resonance wavelength (the Branch_1_ resonance exhibits a singularity as in Fig. 2c,d). It can be seen that the current flows along the two arms are identical and are repelled towards the edges of the SRR arms due to charge repulsion by Coulomb forces.

To assure that Eq. (1) has nontrivial solution, the value of its coefficient matrix M must satisfy the condition |*M*| = 0. Therefore, by setting initial Fano resonances F1 and F3 at wavelengths of 6.36 and 3.86 µm, and tuning initial Fano resonance F2 through varying H_Arm2_ from 0.2 to 1.8 μm (H_Arm1_ is fixed at 1.2 μm), the eigen-frequencies, corresponding to the final resonance wavelengths resulted from the interaction among the initial triple Fano resonances, can be successfully resolved based on Eq. (1) and |*M*| = 0. As shown in Fig. 4a, the relationship between the resulted resonance wavelengths and the height of Arm_2_ exhibits the double anticrossing behaviors, agreeing very well with the numerical calculations in Fig. 2d. It should be mentioned that the singularity in anticrossing is not observed with this oscillator model since the eigen-frequencies merely predict the resonance wavelengths (resonance amplitudes are not included) and there is no symmetry-related factor included in Eq. (1). Nevertheless, both numerical and theoretical results clearly prove the double anticrossings and strong coupling behaviors in the triple Fano resonances.

### Analysis and discussions

For a better understanding of the double Rabi splittings of the Fano resonances, the electric charge distributions of the aSRRs with different arm heights are further simulated using CST Microwave Studio (see Methods). Firstly, the current flow of the aSRRs with H_Arm1_ = 1.2 μm and H_Arm2_ = 0.8 μm are simulated at corresponding initial Fano resonances, respectively, which do not interact with each other since they are away from the anticrossing regions. As shown in Fig. 4b,c, the current flows at Fano resonances F1 and F2 agree well with the schematic plots of the current oscillation modes in the left of Fig. 3a,b, where one arm of the aSRRs supports single resonance in each case. When two Fano resonances are tuned closely to interact, for example when H_Arm1_ = 1.2 μm and H_Arm2_ = 1.1 μm, Fano resonances F1 and F2 couples strongly and the anticrossing occurs (as in Fig. 1c), where the current oscillations are activated along both arms of the aSRRs (Fig. 4d–e). Meanwhile, at the anticrossing region, the current flows at the two branch wavelengths show entirely different distributions: for the upper band (Branch_1_), the current oscillations along the two arms are out-of-phase, i.e. currents flow in the opposite direction (Fig. 4d); while for the lower band (Branch_2_), the current oscillations along the two arms are in-phase, i.e. currents flow in the same direction (Fig. 4e). Therefore, it can be said that it is the interaction between current oscillations that results in the anticrossings, where the circular current flows between the two arms build up the interaction channel to bridge the two resonance modes.

In comparison, when H_Arm1_ = H_Arm2_ = 1.2 μm (corresponding to the singularity position in Fig. 2c,d), the current oscillations are degenerated into a single type as plotted in Fig. 4f, where only the in-phase current oscillation exists and the circular current flow (the interaction channel) vanishes. The fundamental reason for the disappearance of the out-of-phase current oscillation (also the appearance of the singularity during F1-F2 anticrossing) is that the two arms of the symmetric SRRs are identical with respect to the direction of the light polarization and so does any electric current excited on them, which makes the charges oscillate in-phase all the time, i.e. the excitation efficiency of the out-of-phase current oscillation is zero. Once the symmetry of the structure is broken, for example when H_Arm1_ = 1.2 μm and H_Arm2_ = 1.3 μm, the out-of-phase current oscillations along the two arms of the aSRRs will be excited again (Supplementary Fig. S7). As a result, the upper branch of the F1-F2 anticrossing will be recovered, which is consistent with the observations in Fig. 2c,d. Furthermore, a close look at Fig. 2c,d could find that no matter the singularity exists or not, the repelling of the lower band away from the original F1 band position (dashed line) is well preserved during the anticrossing. This is due to the fact that for the in-phase current oscillations, the electric charges on the symmetric SRR arms will push each other away from the arms due to the Coulomb forces, which results in most of the charges distributed along the outer edges of the SRR arms (Fig. 4f). It is this repelling in electric currents that results in the level repulsion in the anticrossing process.

### Experimental demonstrations

**Figure 5:**
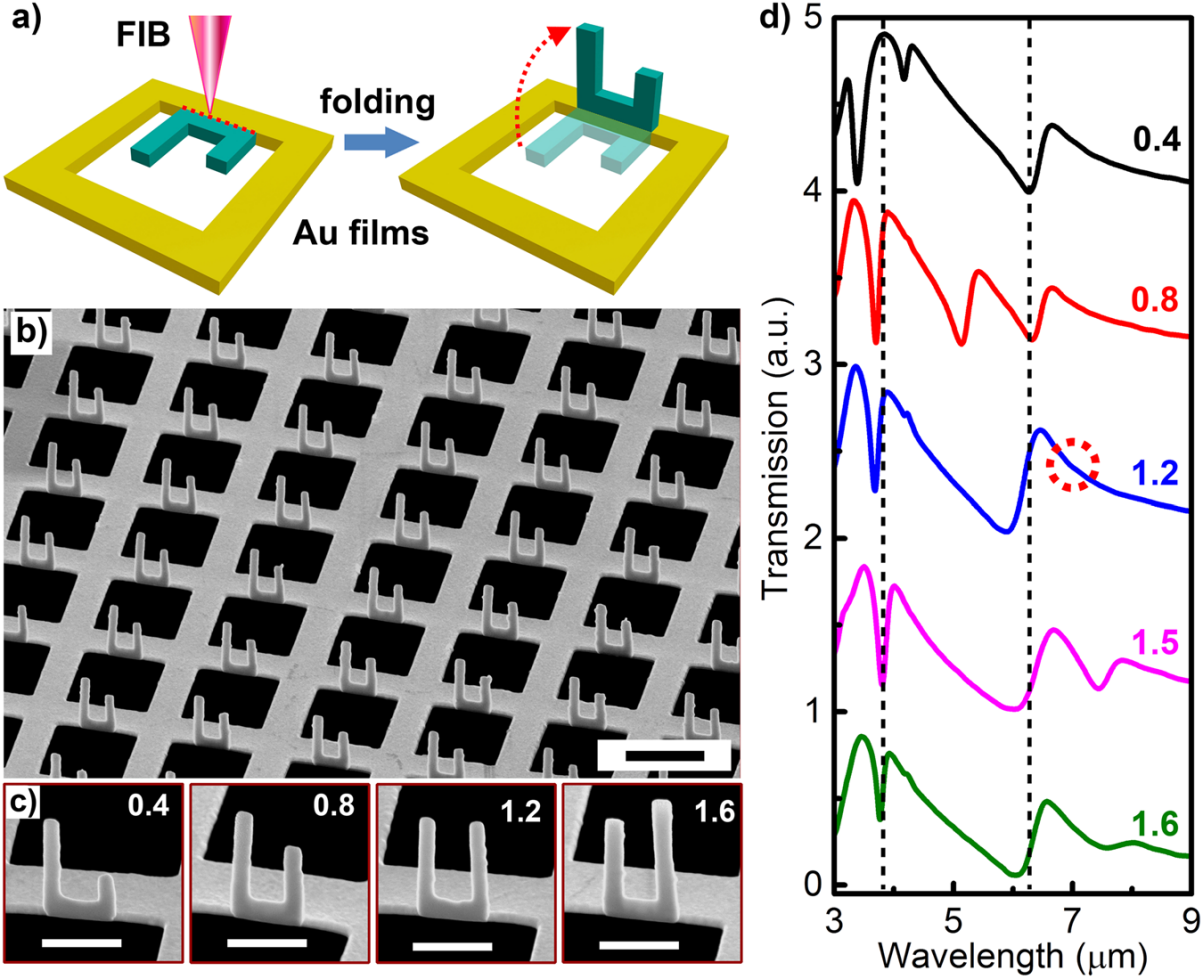
Experimental demonstrations. (**a**) Schematic of the fabrication process with a home-developed ion-beam irradiation-induced folding technique. (**b**) Side-view SEM images of fabricated 3D structures with H_Arm1_ = 1.2 µm and H_Arm2_ = 0.8 µm. Scale bar: 2 µm. (**c**) Side-view SEM images of fabricated aSRRs with different H_Arm2_ as noted while H_Arm1_ is fixed at 1.2 µm. Scale bars: 1 µm. (**d**) Measured transmission spectra of the fabricated structure with H_Arm1_ = 1.2 µm and different H_Arm2_ as noted. Each spectrum from bottom to top is vertically shifted by one unit compared to that below it. The vertical dashed curves denote the initial positions of F1 and F3, respectively, where F2 is tuned across. The dashed circles indicates the singularity in F1-F2 anticrossing when H_Arm1_ = H_Arm2_ = 1.2 μm. Other structural parameters are the same as in Fig. 2.

The 3D plasmonic structures with aSRRs could be readily fabricated by using an *in situ* focused ion beam (FIB) irradiation-induced folding technique that we recently developed^26^. In this method, planar aSRRs are firstly cut by FIB lithography on a free-standing Au metal film (80 nm in thickness) while the bottom part of the aSRRs is still connected to the master film (Fig. 5a). Secondly, the ion beam focus spot is continuously scanned along the bottom edge of the aSRRs, which folds the aSRRs naturally due to the stress induced by ion implantation during scanning. The folding angle is increased with the accumulated dose of ion irradiation through increasing exposure time or ion-beam current^26^. With an ion dose of ~3.2 × 10^7^ ions/μm, a maximum folding angle of 90° could be realized, which enables the realization of vertical aSRRs standing onto the square air hole array perforated in the metallic film (Fig. 5b). Figure 5c shows the side-view SEM images of the fabricated vertical aSRRs with one arm height (H_Arm1_) fixed at 1.2 µm while another arm height (H_Arm2_) is varied from 0.4 to 1.6 µm, respectively, revealing the high-quality replica of the structural designs.

More importantly, the transmission spectra of the fabricated 3D structures in Fig. 5d, measured by a Fourier-transform infrared spectrometer (FTIR)^26^, clearly show the tuning of Fano resonance F2 when the height of Arm_2_ is varied. Meanwhile, double anticrossing behaviors (F1-F2 and F2-F3 anticrossings) are observed during this tuning process, which are well consistent with that depicted in Fig. 2c. As a result, the measured relationship between the resonant wavelengths and the height of Arm_2_ agrees excellently with both numerical calculations and theoretical modeling (Supplementary Fig. S8), as plotted in Fig. 4a. Moreover, the singularity in F1-F2 anticrossing is clearly observed when H_Arm1_ = H_Arm2_ = 1.2 μm, as noted by the dashed circle in Fig. 5d. This nearly perfect matching among numerical, theoretical, and experimental observations unambiguously verify the double Rabi splittings in plasmonic Fano resonances that have not been achieved in previous plasmonic or atomic systems, illustrating the unique advantages of the proposed 3D plasmonic structures in engineering multiple interference states towards strong interactions.

## Discussion

In summary, we have demonstrated the observation of Rabi splitting of two strongly coupled surface plasmon Fano resonance states in an asymmetric SRR based 3D plasmonic structure. The plasmonic system stably supported triple Fano resonance states, arising from the coupling between the broadband planar air hole modes and the narrowband vertical aSRR modes. Strong double anticrossings in Fano resonances have been observed with rigorous numerical simulations, which were excellently explained by an analytical three-oscillator model and successfully demonstrated by experiments with a FIB-induced folding technique. The consistent numerical, theoretical, and experimental observations clearly demonstrated the existence of Rabi splittings in Fano resonances, as well as the unique advantage of the 3D plasmonic system in readily engineering multiple strong couplings. Perhaps due to lack in physical insights and difficulty in fabrication technology, such a strong coupling induced Rabi splitting in Fano resonances, which is a bridge connecting the two prominent fundamental physical phenomena, has not been found in previous atomic and optical systems. The current studies could offer a stimulating insight and a new methodology to explore new fundamental physics, e.g., Fano resonance Rabi splitting, in quantum systems as atoms, molecules, quantum dots, etc. Moreover, the extremely strong coupling effect involved in the Fano resonance Rabi splitting in optical and plasmonic systems could be used to significantly enhance light-matter interaction for high-sensitivity optical sensing and detecting^24^, as well as extend the concept to other objects of interests like mesoscopic quantum point contacts^25^ and phonons^39^.

## Methods

### Numerical simulations

The transmission (T), reflection (R) and E-field distributions of the structures were simulated by using the finite-difference time-domain (FDTD) method. Absorption (A) of the structure was calculated by A = 1-T-R. The current distributions of the structures were calculated by employing the commercial software package CST Microwave Studio^26, 30^. More details about the numerical methods were described in refs 26, 30.

### Sample fabrications

The 3D structures were experimentally fabricated on self-supporting Au films by employing a focused-ion-beam (FIB) irradiation-induced folding technique^26, 30^. Specifically, the pre-designed patterns were firstly cut by utilizing a dual beam FIB/SEM system (FEI Helios 600i). Subsequently, the FIB was continuously scanned using the line scan mode along the bottom edge of the patterned structures, which folded the structures naturally by utilizing the ion-implantation induced stress. During fabrications, the acceleration voltage of Ga^+^ was set to 30 kV and an ion-beam current of 40 or 80 pA was used, with which a maximum folding angle of 90° could be achieved with an accumulated ion dose of ~3.2 × 10^7^ ions/μm^26^. Due to the large scale of the structure, the fabrication resolution is about 20 nm.

### Optical characterizations

Transmission spectra of the structures were measured by using a Fourier-transform infrared spectrometer (Vertex 80, Bruker), equipped with an optical microscope (Hyperion 2000, with a × 15 and 0.4 numerical aperture reflective objective lens)^26^. A homemade aperture was inserted after the reflective objective lens to confine the illumination cone with a spatial size of ~40 µm × 40 µm and a conical angle of ~5°. During measurement, the samples were tilted correspondingly to obtain normal incidence. All transmission spectra were calibrated using air as a reference.

## Electronic supplementary material

Supplementary Information
