# Supplementary material for: Fano resonance Rabi splitting of surface plasmons
Source: Sci Rep. 2017 Aug 14;7:8010. doi: 10.1038/s41598-017-08221-5 (PMC5556087; doi:10.1038/s41598-017-08221-5)
Supplement: Supplementary file 1 — Supplementary Information [file 41598_2017_8221_MOESM1_ESM.pdf]

Supplementary Information for

Fano resonance Rabi splitting of surfaces plasmons

Zhiguang Liu,[1,4,#] Jiafang Li,[1,#,*] Zhe Liu,[1,#] Wuxia Li,[1] Junjie Li,[1] Changzhi Gu,[1,2] and Zhi-Yuan Li[3,1,*]

[1]*Institute of Physics, Chinese Academy of Sciences, Beijing 100190, China*

[2]*Collaborative Innovation Center of Quantum Matter, Beijing 200092, China*

[3]*College of Physics and Optoelectronics, South China University of Technology, Guangzhou 510640, China*

[4]*University of Chinese Academy of Sciences, Beijing 100049, China*

The Supplementary Information includes the following contents:

I. Numerical observation of Fano resonances and Rabi splittings

II. Oscillator modeling of Fano resonances

III. Three-oscillator modeling of Fano resonance Rabi splitting

IV. Simulated current distributions of the 3D plasmonic structure

V. Consistence among simulated, theoretical and experimental data

VI. Influence of the folding angle on Rabi splitting

[#] These authors contributed equally to this work.

*Corresponding authors: jiafangli@aphy.iphy.ac.cn, phzyli@scut.edu.cn

I. Numerical observation of Fano resonances and the Rabi splittings

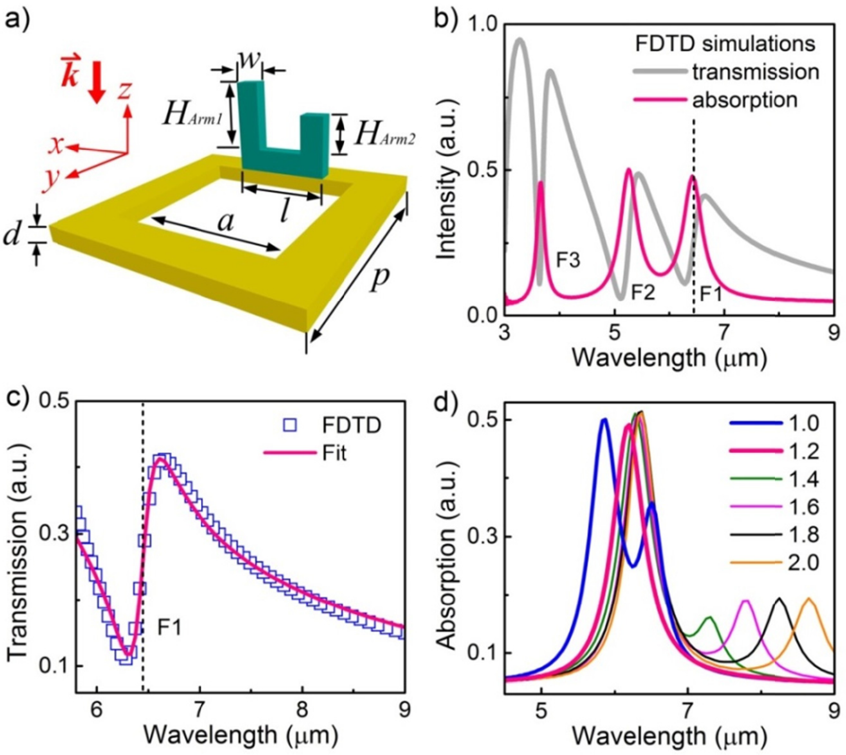

Figure S1. (a) Schematic of the unit cell of the substrate-free 3D plasmonic structure made of gold. Geometrical parameters of the asymmetric split-ring resonators (aSRR) and metallic hole are as labeled. **(b)** Transmission and absorption spectra of the proposed 3D structure with arm height of H_{Arm1}=1.2 μm and H_{Arm2}=0.8 μm. **(c)** FDTD simulated and fitted transmission spectra of Fano resonance F1. Dashed lines in **(b)** and **(c)** denote the center wavelength of the Fano resonance obtained from the fitting, which corresponds well to the absorption peak of F1. **(d)** Absorption spectra of the proposed 3D structure with H_{Arm1}=1.2 μm and different H_{Arm2} as noted in the F1-F2 anticrossing region, where the resonance wavelengths are more clearly identified than the transmission spectra in Fig. 2c. Therefore, to clearly reveal the Rabi splitting of the Fano resonances, absorption spectra with flat background are used in Fig. 2d. Other structural parameters: lattice periodicity *p*=3 μm, square hole width *a*=2 μm, Au film thickness *d*=80 nm, width of SRR *l*=1 μm, and SRR arm width *w*=0.25 μm.

The proposed 3D plasmonic structure is depicted in Fig. S1a, with which three pronounced resonances in both transmission and absorption are clearly observed in Fig. S1b. To verify their nature of Fano resonances, the simulated spectra are fitted using a Fano resonance formula that is based on the typical description [1]:

$$\mathrm{T}(\omega) = \mathrm{T}_0 + C_0 \times \frac{(\Gamma\gamma + \omega - \omega_r)^2}{(\omega - \omega_r)^2 + \gamma^2} \qquad (1)$$

where ω_r ($\omega_r = 2\pi/\lambda_r$) and γ are standard parameters that denote the position and width of the resonance, respectively; Γ is the Fano asymmetry factor. Due to the influence of multiple resonances including the extraordinary optical transmission (EOT) peak of the planar air holes, the transmission spectrum in Fig. S1b cannot be fitted by simply using Eq. (1). For simplicity, the part of Fano resonance F1 is merely taken out and fitted with Eq. (1). As shown in Fig. S1c, the simulated spectrum is well fitted with a Fano asymmetry factor of $\Gamma = -0.83$. The center wavelength of the fitted Fano resonance F1 is identified at $\lambda_r = 6.44$ μm, which is at neither the transmission peak nor the transmission dip but corresponds to the absorption peak at F1 resonance, as noted by the dashed line. Therefore, to clearly reveal the Fano resonance Rabi splittings, absorption spectra with flat background are used in Fig. 2d . Specifically, for the structure with H_{Arm1}=1.2 μm and H_{Arm2}=0.8 μm, the full width at the half maximum (FWHM, Δλ) of the absorption peak ($\lambda_r = 6.437$ μm) is 0.4824 μm. While for the structure with H_{Arm1}=H_{Arm2}=1.2 μm, Δλ is 0.603 μm at $\lambda_r = 6.136$ μm, of which the absorption peak intensity and the relative absorption band ($\Delta\lambda/\lambda_r$) are increased by 3% and 31%, respectively.

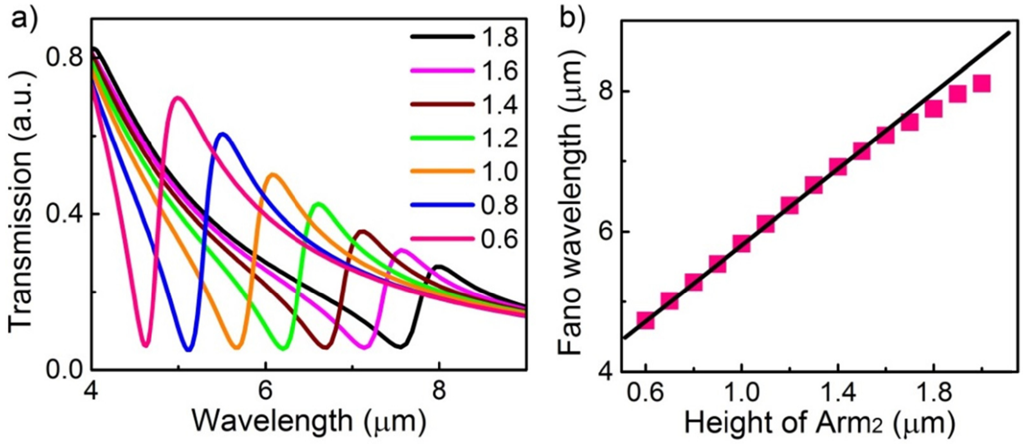

Figure S2. Tunability of Fano resonance F2. (a) Simulated transmission spectra of the 3D structure with H_{Arm1}=0 and different H_{Arm2} (from 0.6 to 1.8 μm) as noted. In such a case, only Fano resonances F2 and F3 can be excited. **(b)** Relationship between Fano resonance wavelength and the height of Arm_2. Solid straight line is the guide to the eye. It can be seen that Fano resonance F2 can be readily tuned by changing the height of Arm_2, which shows a nearly linear relationship with a small deviation at higher Arm_2 (H_{Arm2}>1.5 μm) due to the phase retardation effects. Other parameters are the same as in Fig. S1.

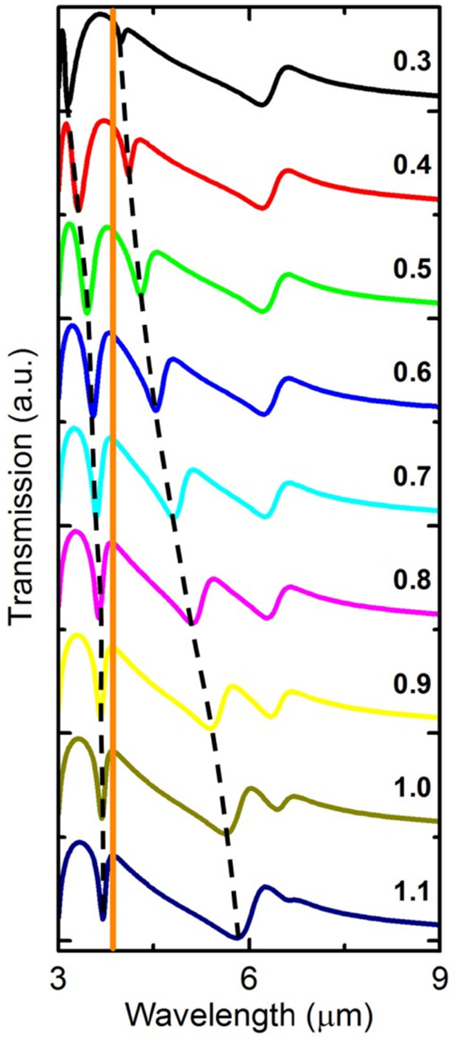

Figure S3. Simulated transmission spectra of the 3D structure with H_{Arm1}=1.2 μm and different H_{Arm2} (from 0.3 to 1.1 μm) as noted. The dashed curves and vertical straight line (guides to the eye) clearly reflect the anticrossing behavior between Fano resonance F2 and F3. Other parameters are the same as in Fig. S1.

One important feature of the proposed 3D plasmonic system is that Fano resonance F2 (or F1) can be readily tuned through varying the height of Arm_2 (or Arm_1), as plotted in Fig. S2, mainly due to the fact that the F2 (or F1) resonance is associated with the in-phase conductive coupled current oscillations along the arm length of the aSRRs (left column of Fig. 3) [2]. This unique property makes possible the proposal in Fig. 1d, i.e. realization of the Fano resonance Rabi splitting through continuous tuning of one Fano resonance, as verified in Figs.

2c-2d and Fig. S3. More importantly, the region of F1-F2 anticrossing is highly dependent on the height of Arm_1. For example, by switching H_{Arm1} from 1.2 to 1.4 μm, the F1-F2 anticrossing position is changed from 1.2 to 1.4 μm, as shown in Fig. S4a. Particularly when H_{Arm1}=0, the double anticrossings degrade into a single anticrossing (F2-F3 anticrossing) since Fano resonance F1 vanishes, as plotted in Fig. S4b.

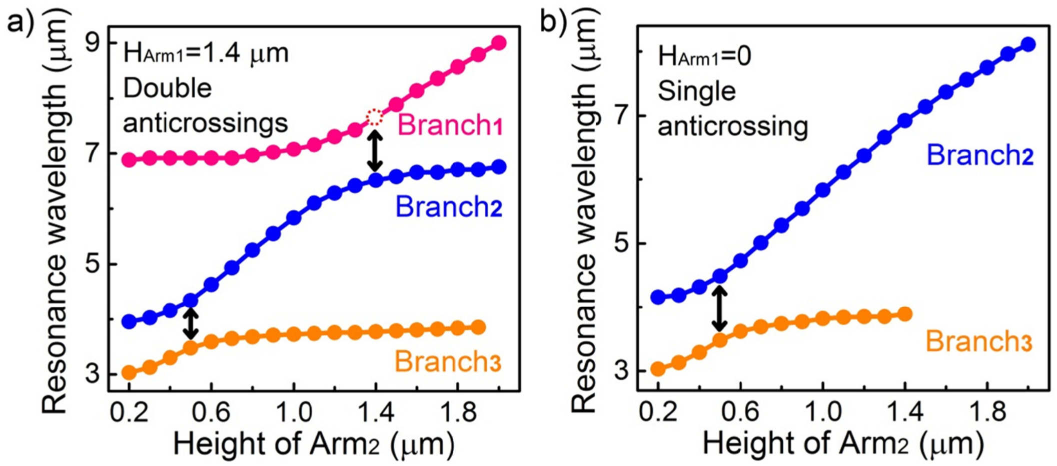

Figure S4. Relationship between the resonance wavelength and the height of Arm_2 under numerical simulations for **(a)** H_{Arm1}=1.4 μm and **(b)** H_{Arm1}=0 μm. The black double-head arrows indicate the position of Fano resonance anticrossing. The dashed circle indicates the singularity in F1-F2 anticrossing when H_{Arm1}=H_{rm2}=1.4 μm. It can be seen that compared with Fig. 2d and Fig. 4a, the F1-F2 anticrossing position can be readily tuned by varying the height of Arm_1. Particularly when H_{Arm1}=0 (Fano resonance F1 disappears), the double anticrossings degrade into a single anticrossing in Fig. S4b. Other parameters are the same as in Fig. S1.

In comparison with Fano resonances F1 and F2, Fano resonance F3 is less tunable and is relative constant around 3.7-3.9 μm when it does not interact with other Fano resonances. This is because at corresponding resonance wavelength, the current density is mainly dominated by the current oscillations along the edge of the planar air hole (as shown in Fig. S5a), although there are two different out-of-phase current oscillations along the two arms of the aSRR. This makes modes M3 and M3' indistinguishable and manifested as one mode

denoted merely by M3, of which the wavelength position is less tunable than M1 and M2. Consequently, the wavelength position of Fano resonance F3, which is induced by the interference between M3 and M0 (as will be illustrated in Fig. S6), is also quite stable. This could also be seen in Fig. S5b, where resonance Branch$_3$ approaches almost the same region (3.7-3.9μm, corresponding to the position of initial F3 resonance) in the cases of H_{Arm1}=0, 1.2, and 1.4 μm. Therefore, the widely tunable Fano resonance F2 is mainly employed as the tuning element in our studies.

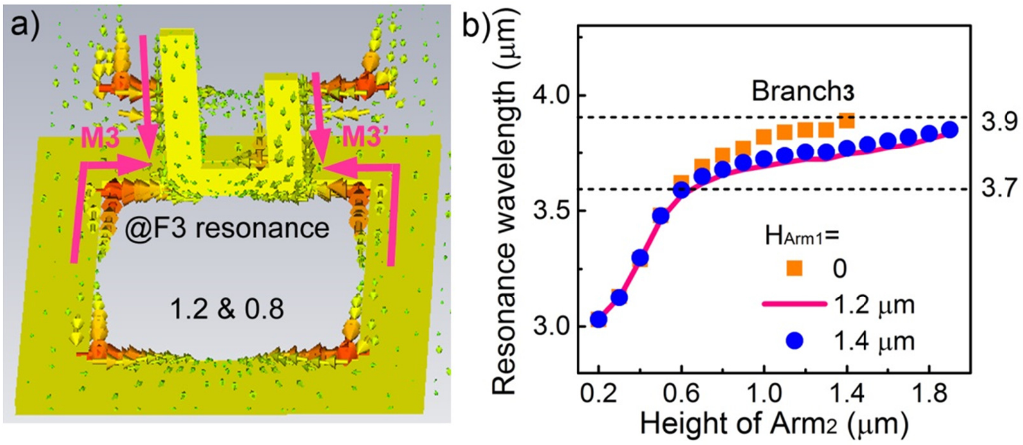

Figure S5. (a) Simulated current distribution of the 3D structure with H_{Arm1}=1.2 μm and H_{Arm2}=0.8 μm at the F3 resonance wavelength. **(b)** Relationship between the resonance wavelength at Branch$_3$ and the height of Arm$_2$ under numerical simulations for H_{Arm1}=0, 1.2 and 1.4 μm, respectively.

II. Oscillator modeling of Fano resonances

One unique feature of the proposed 3D plasmonic system is that it simplifies the interaction among three Fano resonances from a complicated six-body system into a four-body case, which could be analyzed by using a mechanical model consisting of four harmonic oscillators (Fig. 3) [3]. In this model, the four oscillators influence each other through six springs with coupling coefficients of k_{01}, k_{02}, k_{03}, k_{12}, k_{13} and k_{23}, respectively, in which $m_1 =$

$m_2 = m_3 = m_4 = 1$ is set for simplicity. In our case, an input harmonic force $\mathrm{F(t) = Ae^{-i\omega t}}$ is employed to drive the oscillator $|\mathrm{M0}\rangle$, which is analogous to the y-polarized light excitation of the EOT resonance. The equations that describe the motion of oscillators can be represented in terms of the shifts x_0, x_1, x_2, and x_3 from their respective equilibrium position, as

$$\ddot{x}_0 + r_0\dot{x}_0 + w_0^2 x_0 - \mathrm{k}_{01}x_1 - \mathrm{k}_{02}x_2 - \mathrm{k}_{03}x_3 = Ae^{-i\omega t} \quad (2)$$

$$\ddot{x}_1 + r_1\dot{x}_1 + w_1^2 x_1 - \mathrm{k}_{01}x_0 - \mathrm{k}_{12}x_2 - \mathrm{k}_{13}x_3 = 0$$

$$\ddot{x}_2 + r_2\dot{x}_2 + w_2^2 x_2 - \mathrm{k}_{02}x_0 - \mathrm{k}_{12}x_1 - \mathrm{k}_{23}x_3 = 0$$

$$\ddot{x}_3 + r_3\dot{x}_3 + w_3^2 x_3 - \mathrm{k}_{03}x_0 - \mathrm{k}_{13}x_1 - \mathrm{k}_{23}x_2 = 0,$$

where r_i (i=0, 1, 2, and 3) is the friction coefficient used to describe the energy dissipation at corresponding initial resonance frequency w_i, and k_{ij} is the coupling coefficient between resonances w_i and w_j. The displacements x_0, x_1, x_2, and x_3 can be expressed to $x_j = P_j e^{-i\omega t}$, where P_j is the amplitude of the resonance w_j and is not relevant to the time factor *t*. The transmission spectra can be calculated through the formula $\mathrm{T} = |(P_0 + P_1 + P_2 + P_3)^2|$.

To calculate the spectra in Fig. 3, we used four resonances modes, i.e. M0, M1, M2 and M3, to represent the initial resonance of the four mechanical oscillators $|\mathrm{M0}\rangle$, $|\mathrm{M1}\rangle$, $|\mathrm{M2}\rangle$, and $|\mathrm{M3}\rangle$, respectively, as plotted in Fig. S6a. The M0 mode represents the broad EOT resonance, while modes M1, M2 and M3 are the three discrete resonances that describe the current oscillation modes induced by 3D conductive coupling [4]. Based on the spectral features in Fig. 2b, the initial resonant wavelengths λ_{0i} of M0, M1, M2 and M3 are set as 3.43, 6.42,

5.18 and 3.63 μm ($w_i = 2 * \pi * c/\lambda_{0i}$, c is the speed of light in a vacuum), respectively, and the friction coefficients r_i are set as $r_0 = 35 * 10^{13}, r_1 = 1.9 * 10^{13}, r_2 = 2.2 * 10^{13}$ and $r_3 = 1.2 * 10^{13}$. One can notice that the friction coefficient of M0, i.e. the continuum state, is over ten times larger than the discrete states (M1, M2 and M3).

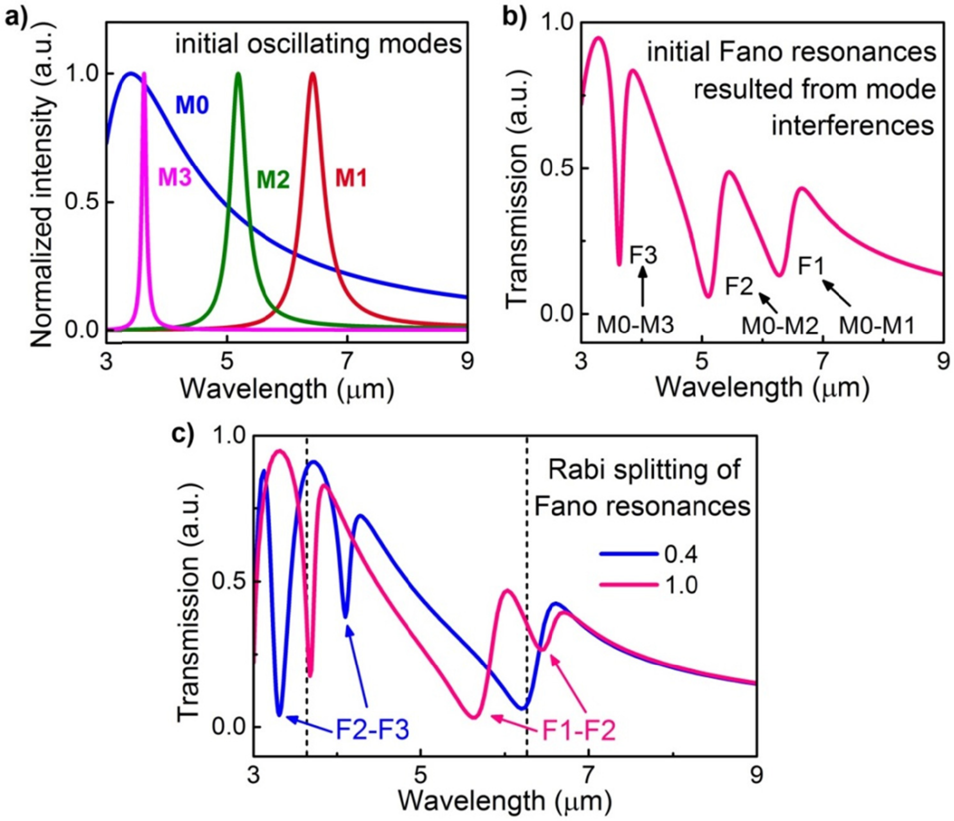

Figure S6. (a) Normalized spectra of four modes M0, M1, M2, and M3 that represent the initial resonance of the four mechanical oscillators |M0⟩, |M1⟩, |M2⟩, and |M3⟩, respectively, in the four-oscillator model. **(b)** The broad resonance M0 serves a "continuum" state that interferes with other three "discrete" states, i.e. narrow resonances M1, M2 and M3, which results in the initial triple Fano resonances F1 (by M0-M1 interference), F2 (by M0-M2 interference), and F3 (by M0-M3 interference). **(c)** Simulated transmission spectra of the structure with different H_{Arm2}=0.4 μm (blue curve) and H_{Arm2}=1.0 μm (red curve). Vertical dashed lines denote the position of the initial Fano resonance F1 and F3, respectively, as in (b). Rabi splittings between Fano resonances can be clearly seen when the initial Fano resonance F2 is tuned closely to the initial F1 (red curve as the F1-F2 interaction) or the initial F3 (blue curve as the F2-F3 interaction) resonance.

In the case of Fig. 3, the triple Fano resonances are far away from the anticrossing regions, i.e. the tripe Fano resonances are relatively independent. In such a specific structure, $\mathrm{k}_{12} = \mathrm{k}_{13} = \mathrm{k}_{23} = 0$ can be used to simply the model. If one further sets $\mathrm{k}_{02} = \mathrm{k}_{03} = 0$ and $\mathrm{k}_{01} \neq 0$ (in all cases, coupling coefficients k_{01}, k_{02} and k_{03} are set to $1.1 * 10^{28}$, $2.7 * 10^{28}$, and $3.2 * 10^{28}$, respectively, when they are not set to zero), the system is then simplified into to a two-oscillator model in Fig. 3a, as

$$\ddot{x}_0 + r_0\dot{x}_0 + w_0^2 x_0 - \mathrm{k}_{01} x_1 = Ae^{-i\omega t} \quad (3)$$

$$\ddot{x}_1 + r_1\dot{x}_1 + w_1^2 x_1 - \mathrm{k}_{01} x_0 = 0.$$

With this equation, the spectrum in the right of Fig. 3a can be calculated, where Fano resonance F1 is reproduced and could be considered as a two-state coupling between the broad EOT resonance M0 and a narrow resonance M1 (resulted from the 3D conductive coupling between Arm1 and the metallic hole), as illustrated in left of Fig. 3a.

Similarly, in the case of $\mathrm{k}_{03} = 0$, $\mathrm{k}_{01} \neq 0$, and $\mathrm{k}_{02} \neq 0$, a three-oscillator system is constructed in Fig. 3b, as

$$\ddot{x}_0 + r_0\dot{x}_0 + w_0^2 x_0 - \mathrm{k}_{01} x_1 - \mathrm{k}_{02} x_2 = Ae^{-i\omega t} \quad (4)$$

$$\ddot{x}_1 + r_1\dot{x}_1 + w_1^2 x_1 - \mathrm{k}_{01} x_0 = 0$$

$$\ddot{x}_2 + r_2\dot{x}_2 + w_2^2 x_2 - \mathrm{k}_{02} x_0 = 0,$$

which generates double Fano resonances (F1 and F2) due to the introduction of the mode M2 associated with the short arm of the aSRRs (Arm_2), as illustrated in the left of Fig. 3b.

In the realistic case, k_{01}, k_{02}, and k_{03} are all non-zero and the plasmonic system should be described by a four-oscillator system that induces triple Fano resonances F1, F2 and F3, as manifested in Fig. 3c. The equations can be presented as

$$\ddot{x}_0 + r_0\dot{x}_0 + w_0^2 x_0 - \mathrm{k}_{01}x_1 - \mathrm{k}_{02}x_2 - \mathrm{k}_{03}x_3 = Ae^{-i\omega t} \quad (5)$$

$$\ddot{x}_1 + r_1\dot{x}_1 + w_1^2 x_1 - \mathrm{k}_{01}x_0 = 0$$

$$\ddot{x}_2 + r_2\dot{x}_2 + w_2^2 x_2 - \mathrm{k}_{02}x_0 = 0$$

$$\ddot{x}_3 + r_3\dot{x}_3 + w_3^2 x_3 - \mathrm{k}_{03}x_0 = 0.$$

With this equation, the spectra in the right of Fig. 3c and Fig. S6b can be calculated, where all the triple Fano resonance F1, F2 and F3 are reproduced and agree very well with the numerical calculation in Fig. 2b, indicating that the triple Fano resonances are indeed induced from the interference between M0 and M1, M2 and M3, respectively. Compared with M1 and M2 that result from the in-phase coupling between the current oscillations on each arm of the aSRR and the edge of planar air hole, M3 is an out-of-phase coupling mode[2, 4], as illustrated in the left of Fig. 3c and Fig. S5a. Although there are two current oscillation modes (M3 and M3') along the two different arms of the aSRRs, they are manifested into one resonance (M3) due to the dominated charges distribution along the bottom edge of the air hole (Fig. S5a).

Now the origins of all the three Fano resonances in the aSRR-based plasmonic system are clear (as illustrated in Fig. S6b): Fano resonance F1 results from the interference between M0 and M1; Fano resonance F2 is formed due to the coupling between M0 and M2; the interaction between M0 and M3 results in Fano resonance F3. It can be seen that for all the three Fano resonances, the broad EOT resonance mode M0 serves as a shared superradiant mode, providing energy to couple with the other three subradiant modes respectively, which is the unique feature of our plasmonic system.

III. Three-oscillator modeling of Fano resonance Rabi splitting

In the anticrossing regions of Fano resonances, the perquisite $\mathrm{k}_{12} = \mathrm{k}_{13} = \mathrm{k}_{23} = 0$ is no longer satisfied. As a result, the non-homogeneous linear differential Eq. (2) becomes complicated when M2 is continuously tuned by varying $\mathrm{H}_{\mathrm{Arm2}}$ (each value of $\mathrm{H}_{\mathrm{Arm2}}$ introduces three extra different coupling coefficients k_{02}, k_{12}, and k_{23}). This introduces difficulties in finding nontrivial solution and eigenvalues of the inhomogeneous linear differential equation. On the other hand, this complicated interaction can be simplified into the interaction purely among the initial triple Fano resonances, i.e. the interaction merely among initial Fano resonance F1, F2 and F3. This simplification is reasonable as the transmission spectra of the 3D plasmonic structure shown in Fig. S6c, where the F1-F2 Rabi splitting results from the interaction between Fano resonances F1 and F2, and F2-F3 Rabi splitting results from the interaction between Fano resonances F2 and F3. Therefore, the interaction among triple Fano resonances can be described by a three-oscillator mode and represented simply by a homogeneous linear differential equation with which eigenvalues could be easily obtained, as

$$\ddot{y}_1 + s_1\dot{y}_1 + f_1^2 y_1 - \Lambda_{12} y_2 - \Lambda_{13} y_3 = 0 \tag{6}$$

$$\ddot{y}_2 + s_2\dot{y}_2 + f_2^2 y_2 - \Lambda_{12} y_1 - \Lambda_{23} y_3 = 0$$

$$\ddot{y}_3 + s_3\dot{y}_3 + f_3^2 y_3 - \Lambda_{13} y_1 - \Lambda_{23} y_2 = 0$$

where $y_i = Q_i e^{-i\omega t}$ (i=1, 2, and 3) represents the resulted resonances, s_i is the friction coefficient, f_i is the frequency of the initial Fano resonance, and Λ_{ij} is the coupling coefficient between Fano resonances f_i and f_j. After replacing the displacements y_j to $Q_j e^{-i\omega t}$, the equations can be expressed in matrix form

$$\begin{pmatrix} -\omega^2 - is_1\omega + f_1^2 & -\Lambda_{12} & -\Lambda_{13} \\ -\Lambda_{12} & -\omega^2 - is_2\omega + f_2^2 & -\Lambda_{23} \\ -\Lambda_{13} & -\Lambda_{23} & -\omega^2 - is_3\omega + f_3^2 \end{pmatrix} \begin{pmatrix} y_1 \\ y_2 \\ y_3 \end{pmatrix} = \begin{pmatrix} 0 \\ 0 \\ 0 \end{pmatrix}. \tag{7}$$

Since the Eq. (7) has nontrivial solution, the value of its coefficient matrix M must meet the condition

$$|M| = \begin{vmatrix} -\omega^2 - is_1\omega + f_1^2 & -\Lambda_{12} & -\Lambda_{13} \\ -\Lambda_{12} & -\omega^2 - is_2\omega + f_2^2 & -\Lambda_{23} \\ -\Lambda_{13} & -\Lambda_{23} & -\omega^2 - is_3\omega + f_3^2 \end{vmatrix} = 0. \quad (8)$$

Therefore, by setting initial Fano resonances F1 and F3 at wavelengths of 6.36 and 3.86 μm, and tuning initial Fano resonance F2 through varying H_{Arm2} from 0.2 to 1.8 μm, the interaction among the initial triple Fano resonances can be successfully resolved based on Eq. (8). As shown in Fig. 4a, the relationship between the resulted resonance wavelengths and the height of Arm_2 clearly exhibits the double anticrossing behavior, agreeing very well with the numerical calculations in Fig. 1d. Here the coupling coefficients are set as $\Lambda_{12} = 0.35 * 10^{28}$, $\Lambda_{13} = 1.2 * 10^{28}$, $\Lambda_{23} = 5 * 10^{28}$.

IV. Simulated current distributions of the 3D plasmonic structure

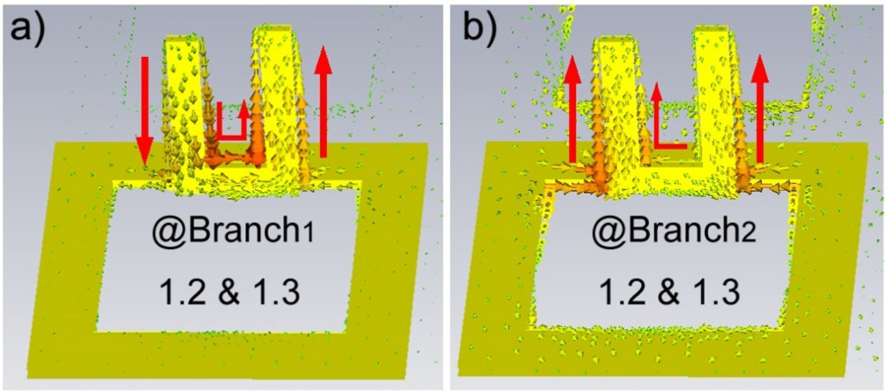

Figure S7. Simulated current distributions of the structure with H_{Arm1}=1.2 μm and H_{Arm2}=1.3 μm at corresponding resonance wavelengths in **(a)** $Branch_1$ and **(b)** $Branch_2$, respectively. It can be seen that once the symmetry of the structure is broken, the out-of-phase current oscillations in **(a)** will be excited again, in comparison with the single type of current flows shown at the singularity point when H_{Arm1}=H_{Arm2}=1.2 μm (Fig. 4f).

V. Consistence among simulated, theoretical and experimental data

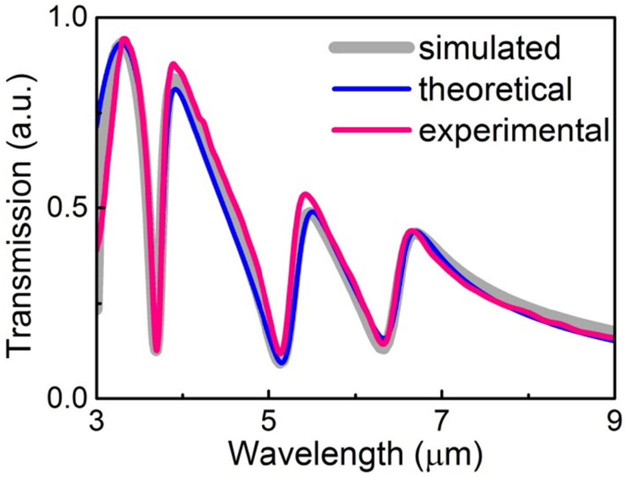

Figure S8. Simulated, theoretical and experimental plots of the transmission spectrum of the proposed 3D structure. Triple Fano resonances are clearly and consistently observed. Due to the realistic fabrication resolution, here the parameters are used with H_{Arm1}=1.2 μm, H_{Arm2}=0.79 μm and a=2.05 μm. Other parameters are the same as in Fig. S1.

VI. Influence of the folding angle on Rabi splitting

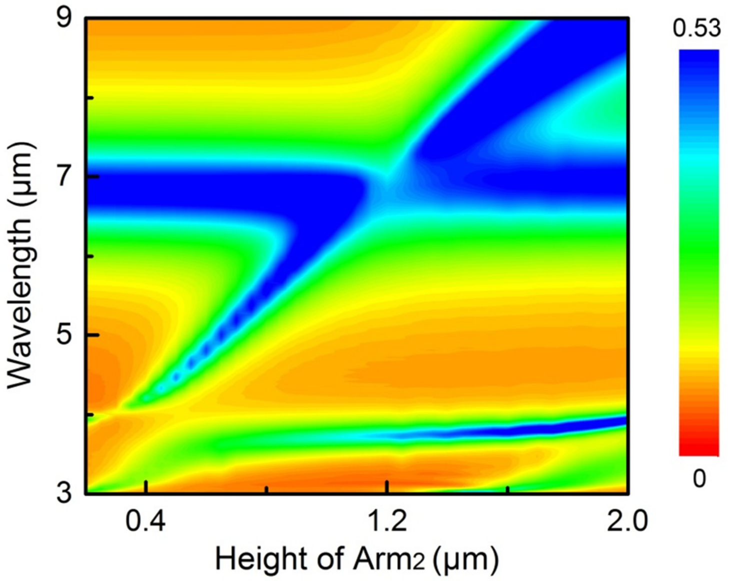

Figure S9. Colormap of the simulated absorption spectra of the proposed 3D structure with folding angle of 60° versus the height of Arm_2. All parameters except the folding angle are the same as those in Fig. 1d.

Similar to the 3D structures with symmetric SRRs[2], the steep profile of the asymmetric Fano lineshape in aSRR-based 3D structures degrades quickly with the decrease of folding angle. As a result, the interaction between Fano resonance drops, which affects the Fano resonance Rabi splitting dramatically. For example, when the inclination folding angle reduces from 90° to 60°, as shown in Fig. S9 (in drastic contrast to Fig. 1d), no visible Rabi splittings are observed.

References:

1. Luk'yanchuk, B. et al. The Fano resonance in plasmonic nanostructures and metamaterials. *Nat. Mater.* **9**, 707-715 (2010).
2. Cui, A.J. et al. Directly patterned substrate-free plasmonic "nanograter" structures with unusual Fano resonances. *Light-Sci Appl* **4**, e308 (2015).
3. Mukherjee, S. et al. Fanoshells: Nanoparticles with Built-in Fano Resonances. *Nano Letters* **10**, 2694-2701 (2010).
4. Liu, Z.G. et al. 3D conductive coupling for efficient generation of prominent Fano resonances in metamaterials. *Sci Rep-Uk* **6**, 27817 (2016).
